# Teleradiology and Emergency Neurosurgery-Presence in a Small Asian City State and Need in a Large Canadian Province

**DOI:** 10.4137/jcnsd.s2216

**Published:** 2009-02-17

**Authors:** Wai Hoe Ng, Ernest Wang, Ivan Ng, Mark Bernstein

**Affiliations:** 1 Department of Neurosurgery, National Neuroscience Institute, Singapore.; 2 Division of Neurosurgery, Toronto Western Hospital.

**Keywords:** teleradiology, telemedicine, neurosurgery emergency, image transfer, MMS

## Abstract

Teleradiology involving the transfer of vital patient information such as scan images is an important technology to facilitate effective and efficient provision of neurosurgical care in the setting of scarce resources and geographic isolation. We review the implementation of teleradiology initiatives in the small city state of Singapore and its potential and need in the large province of Ontario and draw parallels in their strategic implementation. Although it may seem intuitive that teleradiology has greater applications in regions of vast geographical size, the technology has universal usefulness if applied appropriately in any neurosurgical or health care system.

## Introduction

Singapore is small city state with a small land area of 707.1 km^2^ located at the southern tip of the Malay Peninsula with a population of approximately 4.84 million.^1^ The vast majority of neurosurgical service is provided by the National Neuroscience Institute (NNI) through 4 campuses located over the island.

Ontario is a huge geographic contrast to Singapore. It is the second largest province in Canada with a land area of 1,076,395 km^2^ and population of 12.8 million.^2^,^3^ The capital of Ontario is Toronto located on the north-western shore of Lake Ontario. Toronto is the largest city in Canada and has a metropolitan population of approximately 5.1 million.^3^

Approximately 7000 neurosurgical cases are operated per year in the four University of Toronto teaching hospitals. Other centers exist in Mississauga (30 km west of Toronto), Hamilton (70 km west), London (150 km west), Kingston (250 km east), and Sudbury (400 km north). Two other centers exist in Ontario more than 500 km from Toronto. The numerous other hospitals without neurosurgical services in Ontario rely on these units for their neurosurgical patients. Essentially all Ontario hospitals are publicly funded.

## Teleradiology in Singapore

Emergency health services in Singapore are largely supported by the public emergency services and public hospitals. The vast majority of emergency neurosurgery lies with the purview of the NNI who is the centralized national provider of neuroscience services for the country.

Neurosurgical emergencies form a significant proportion of overall workload at the NNI and the provision of this essential service operates on a two-tier system after standard working hours. The in-house service is provided by a neurosurgeon-in-training (registrar or resident) with a senior staff (attending or consultant) who is normally off-site but contactable via telephone consultation and will return to the hospital for any problems arising that may require more senior supervision or expertise. This two-tier system is a common practice in many parts of the world.

Reliance solely on verbal information conveyed via telephoned referrals have limitations in the quality of information provided which translates to impairment in patient assessment and management.^4^

Accurate scan interpretation is arguably the most crucial diagnostic tool for neurosurgical emergencies. The complexity of neuroanatomy often mandates a steep learning curve and this is further compounded by the small margin of error and time critical nature of a neurological emergency that warrants prompt and accurate diagnosis for the emergent institution of definitive therapy.

**Figure 1:**
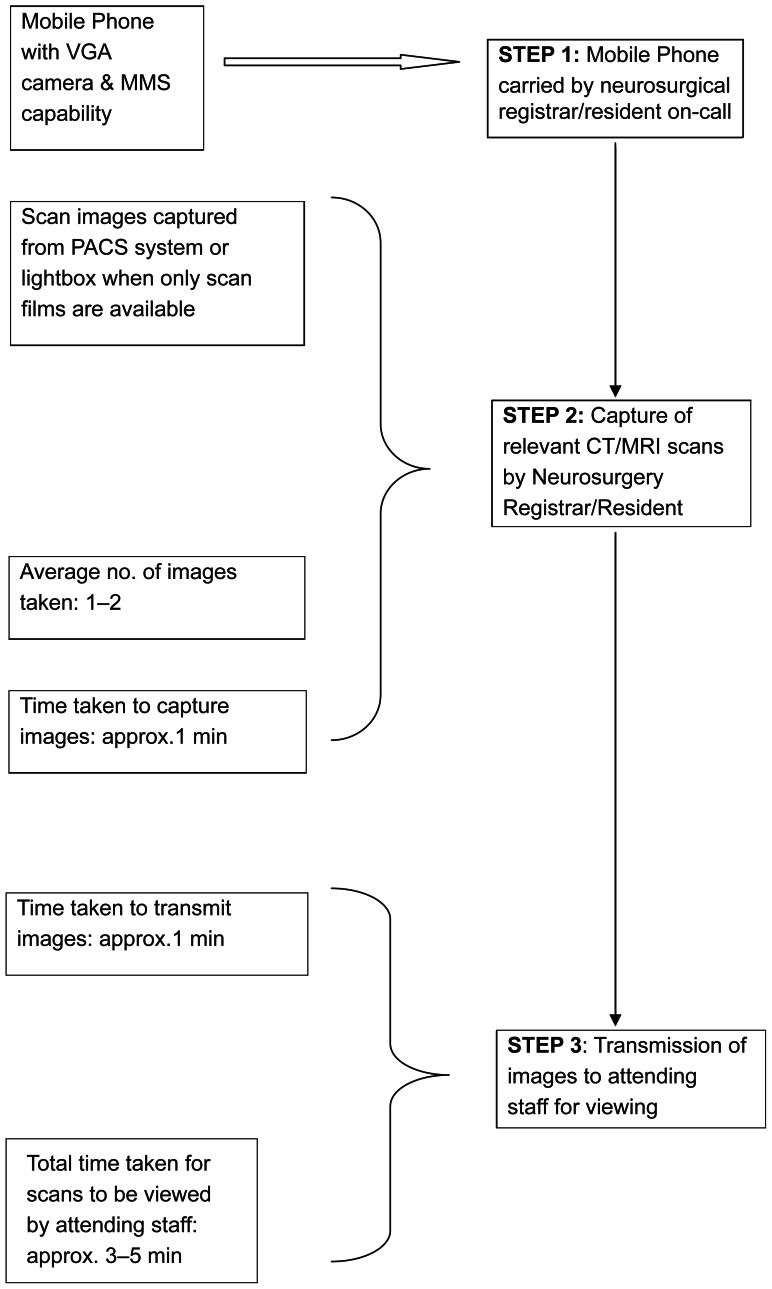
Workflow for MMS teleradiology.

Since 2006, we have introduced the use of Multimedia Messaging Service (MMS) teleradiology at our service to bridge the deficiencies in scan interpretation.^5^,^6^ The in-house staff is provided with a mobile phone with Video Graphics Array (VGA) camera and MMS capabilities. All attending consultant staff have personal MMS-enabled mobile phones. When faced with a clinical scenario, the in-house staff takes one or two representative images off the computer console of the Picture Archival and Communication System (PACS) with the VGA camera and transmits it to the attending staff. In the rare instance when only hard copies of the scans are available, the images are taken off the scans put up on a standard X-Ray viewing lightbox. A standard phone consult will then take place between the resident and the attending staff with exchange of important clinical information. A decision is then taken on the best management course for the patient. The workflow is summarized in Figure 1.

The choice of MMS over other image transfer systems such as email and Integrated Service Digital Network was made based on its cost-effectiveness, ready availability, ease of implementation and portability. The major advantage of the MMS teleradiology system is the ability to visualize high quality images through the mobile phone anywhere without the need for a computer workstation.

One year review after implementation of the program in the form of a standardized questionnaire showed that the technology was well utilized. The questionnaire also demonstrated that the senior staff felt more confident in making critical clinical decisions over the telephone with the aid of the MMS images and junior residents benefited significantly from the input from the senior staff. The images were also deemed to be of sufficiently high quality and it was noteworthy that that viewing the full complement of scans the next day would not have significantly altered clinical decision-making. It is now three years since the implementation of the system and the system is now very much entrenched in the emergency evaluation system of the unit. With increasing experience, the on-call staff is now able to transmit one or two of the most representative images compared to three or four images when the project was first implemented. This has effectively reduced the transmission time of images from three to five minutes to approximately two to three minutes. In-house staff also frequently type out a short text message with the transmitted images to provide a concise summary of the clinical status of the patient. The fact that the technology has been embraced so readily is testimony to its seamless integration and ease of use.

In the near future, the NNI will likely provide neurosurgical service provision to a fifth campus and there are plans to link up the radiology systems via the MMS system or in the form of a digital imaging and communication in medicine (DICOM) e-mail system.^7^

## Need for Teleradiology in Toronto and Ontario

Ontario is a large province and neurosurgery is regionalized to several centres that serve a population which is dispersed over a vast geographic region where there is no access to neurosurgical service. However in spite of the fact that neurosurgical centres are located in relatively few cities, CT and MRI facilities are readily available in many centres without neurosurgical support and thus it is necessary to be able to electronically transfer the images.^8^

Criticall is a central triage system within the province. An emergency physician 1000 km away simply calls one phone number and then an assistant calls around the province to the neurosurgeons on call until the patient’s needs are met. There are three categories of such Criticall referrals for the neurosurgeon on-call. First, there are patients who clearly need immediate transfer (e.g. a young person with traumatic quadriplegia; an adult with acute subdural hematoma and GCS 8). At the other end of the spectrum are patients who can clearly be cared for by the local hospital after phone consultation (e.g. a known tumour patient with a seizure and no new pathology on imaging; a 90 year old hypertensive with a basal ganglia hemorrhage). In the middle are patients who may need neurosurgical consultation in a semi-urgent manner within hours or a day or so (e.g. a patient with possible cauda equine syndrome and a mid-sized disc herniation on MRI); a patient with a good-grade subarachnoid hemorrhage).

At the University Health Network (UHN), the largest neurosurgical unit in Ontario, there are about two emergency neurosurgical patients transferred via Criticall per day and up to twenty or more other calls the neurosurgeon on-call must field.

In all these scenarios and phone consultations, neurosurgeons are currently making decisions without the benefit of being able to look at the imaging and must rely on the verbal description of CT and MRI scans by emergency physicians or general radiologists, as there is no teleradiology system available in this vast province as of yet. It is not feasible, safe, or affordable to have all such patients physically transferred to the closest neurosurgical unit just for a consultation with the imaging present.

Within the hospital setting, many neurosurgeons can access imaging at home by connecting to their hospital desk top with a “Mobikey” device and access the hospital PACS system and discuss the case on the phone with the resident while studying the imaging. This however only applies to patients who are physically within the hospital. The two-tier system of in-house trainees communicating with staff after hours is similar to that described in Singapore.

Neurosurgery is a specialty highly reliant on imaging. Tele-imaging is of strategic importance for the modern practice of neurosurgery in areas where there is little or no access to neurosurgical expertise. It is therefore timely that the province of Ontario has just recently approved resources for infrastructure and technology to create a province-wide teleradiology system by the end of 2009.

## Discussion

Dilemmas in medicine in general and specifically neurosurgery are surprisingly similar even in very different social and cultural contexts. The main issues in emergent neurosurgical management are (a) the need for prompt imaging and accurate interpretation and (b) proper evaluation to assess the need for patient transfer to a dedicated neurosurgical service.

Brain imaging in the form of a CT or MRI scan is the single most important investigation in neurosurgery and its accurate interpretation cannot be overemphasized. Interhospital transfer frequently over long distances carries many potential hazards. This problem was highlighted in the 1980s by Gentleman and Jennett who reported a relatively high proportion of head injury patients who died enroute to the neurosurgical unit.^9^ Moreover, unnecessary transfers will add further financial and logistic stress to the healthcare system and contribute to injudicious utilization of valuable resources. The Norwegian experience has demonstrated that teleradiology can avoid unnecessary patient transfer, alter the treatment at the referring hospital and facilitate emergent transfer to a significant degree.^10^

The use of teleradiology in neurosurgery has been shown to have great benefit in many countries with unique and different socioeconomic, infrastructural and geographical profiles. Examples of its use and validation have been demonstrated in Germany, Ireland, Hong Kong, Israel, France, Poland, Spain, Norway, United Kingdom and South Africa.^10^–^21^ Furthermore, this technology has also been applied successfully to other fields of medicine such as emergency medicine, radiology, orthopaedic surgery, urology and even veterinary medicine.^22^–^26^

In our review on the application of teleradiology in Singapore and Ontario-two regions in the world on extreme ends of the scale in terms of geographic size, it may seem ironic that a small city state with rapid and easy access to neurosurgical services has embraced the use of teleradiology sooner than a much larger and populous province like Ontario. Yet when one delves deeper, the same management issues and constraints confront both Singapore and Toronto neurosurgeons and the possible solutions are perhaps not too surprisingly different. For instance, the MMS system is a simple, cost-effective and can be easily implemented in Ontario so that attending neurosurgical staff in Toronto are able to visualize critical scan images transmitted to their mobile phones from a remote regional hospitals.

Technology is progressing at a relentless pace with increasingly more powerful computers, sophisticated computer networks, superior optics and imaging devices. The limits on applications of Telemedicine will not be bound by technology and the onus now lies with neurosurgical and medical fraternity at large to capitalize on the vast available resources at our disposal.
